# Noise pollution limits metal bioaccumulation and growth rate in a filter feeder, the Pacific oyster *Magallana gigas*

**DOI:** 10.1371/journal.pone.0194174

**Published:** 2018-04-04

**Authors:** Mohcine Charifi, Alison Miserazzi, Mohamedou Sow, Mickael Perrigault, Patrice Gonzalez, Pierre Ciret, Soumaya Benomar, Jean-Charles Massabuau

**Affiliations:** 1 University of Bordeaux, EPOC, Arcachon, France; 2 CNRS, EPOC, Talence, France; 3 Unit of Research on Biological Rhythms, Neuroscience and Environment, Faculty of Science, Mohammed V-Agdal University, Rabat, Morocco; Bigelow Laboratory for Ocean Sciences, UNITED STATES

## Abstract

Shipping has increased dramatically in recent decades and oysters can hear them. We studied the interaction between noise pollution and trace metal contamination in the oyster *Magallana gigas*. Four oyster-groups were studied during a 14-day exposure period. Two were exposed to cadmium in the presence of cargo ship-noise ([Cd^++^]w ≈ 0.5 μg∙L^-1^; maximum sound pressure level 150 dBrms re 1 μPa), and 2 were exposed only to cadmium. The Cd concentration in the gills ([Cd]g) and the digestive gland ([Cd]dg), the valve closure duration, number of valve closures and circadian distribution of opening and closure, the daily shell growth-rate and the expression of 19 genes in the gills were studied. Oysters exposed to Cd in the presence of cargo ship-noise accumulated 2.5 times less Cd in their gills than did the controls without ship noise and their growth rate was 2.6 times slower. In the presence of ship noise, oysters were closed more during the daytime, and their daily valve activity was reduced. Changes in gene activity in the gills were observed in 7 genes when the Cd was associated with the ship noise. In the absence of ship noise, a change in expression was measured in 4 genes. We conclude that chronic exposure to cargo ship noise has a depressant effect on the activity in oysters, including on the volume of the water flowing over their gills (Vw). In turn, a decrease in the Vw and valve-opening duration limited metal exposure and uptake by the gills but also limited food uptake. This latter conclusion would explain the slowing observed in the fat metabolism and growth rate. Thus, we propose that cargo ship noise exposure could protect against metal bioaccumulation and affect the growth rate. This latter conclusion points towards a potential risk in terms of ecosystem productivity.

## Introduction

Aquatic animals are subjected to numerous sensory inputs, including biological and physical effects of natural and anthropogenic origins. Unlike chemical and visual stimuli, sound is transmitted in water over several kilometers with less attenuation [1]. Sound is biologically essential to marine animals [2–5], but noise pollution is a growing problem because it interferes with the normal sound landscape. Indeed, in recent decades, human activity in the ocean has increased considerably along with economic growth [6], causing an increase in the level of ocean ambient noise [7]. For example, the noise pollution caused by shipping results in an increase in the level of ambient noise at an average rate of approximately ½ dB per year since the 1950s [8,9]. Shipping is now largely recognized as a major source of pollution and has been introduced as one of the 11 descriptors of the Marine Strategy Framework Directive (MSFD) to achieve a Good Environmental Status (GES).

Several studies have reported on the adverse effects of noise pollution in marine animals. Noise pollution can have different effects (see Peng et al. [10] for a review), depending on the intensity and frequency of the transmitted noise and the distance of the animal from the source. However, a great gap in our understanding still exists on the effects of noise on marine animals, especially in fish and invertebrates [11].

The small amount of studies on the impact of noise pollution on invertebrates [12–14], especially on bivalve mollusks, does not in any way reflect the importance of invertebrates to the ecosystem. Mosher [15] was probably the first who published on sound detection in bivalve mollusks. He reported an induced burrowing behavior in the Baltic clam or Baltic tellin *Macoma balthica* when the wall of its experimental tank was stimulated with 2–50 Hz. Then, Ellers [16] studied the swash-riding clam and demonstrated its ability to detect vibrations produced by the waves on sandy beaches. More recently, while studying the blue mussel *Mytilus edulis*, Roberts et al. [17] examined the impact of substrate-borne vibrations and demonstrated the sensitivity of the mussel to a range of low frequencies. Solan et al. [18] reported that a noise mimicking offshore shipping and construction activity may alter the mediating contributions made by sediment-dwelling invertebrates in the process of nutrient cycling. They reported that additional noise causes behavioral disturbances and a reduction in surface relocation activity. In another study on the razor clam, Peng et al. [19] detected variation in the burrowing behavior and the expression of metabolic genes in response to a change in the noise intensity from 80 to ≈100 dB re 1 μPa. Such a change causes the clam to escape deep into the mud and enter a state of inactivity. Finally, Charifi et al. [20] described the capacity to hear in the Pacific oyster *Magallana gigas* (formerly *Crassostrea gigas*) and discussed the role this response could play in the lives of these oysters today. Based on the above dataset showing how anthropogenic noise can shape bivalve behavior, we hypothesized that this noise could also induce changes in the volume of the water flow during ventilation and thus interfere with the contaminant bioaccumulation process and/or other physiological mechanisms. The aim of the present report was to test this hypothesis in the widely distributed Pacific oyster *M*. *gigas*.

*Magallana gigas* (Thunberg, 1793) is a common oyster species that has been introduced extensively around the world for aquacultural purposes. It is a filter-feeding animal of major economic interest and often is used as a bioindicator of the state of the marine environment [21]. The present study investigated the effect of noise pollution and its interference with metal pollution. Behavioral, ecophysiological, and genic analyses were conducted because, when considered in combination, they may provide larger insights into the perturbation of the organism.

Animals were exposed to cadmium to obtain insights, on the one hand, of the cellular impact of the metal in the presence and absence of cargo ship noise and, on the other hand, as an indirect marker of ventilatory activity. Indeed, when a constant concentration of trace metal is maintained in the water, variation in ventilatory activity is a limiting step that acts on metal bioaccumulation in fish, mollusks, and crustaceans [22–28]. A high ventilatory flow in the gills strongly enhances the accumulation of Cd in the soft tissues and vice versa. In shrimps, changes in the ventilatory activity modulates the Cd bioaccumulation, but [Cd]w alone does not influence ventilatory and cardiac frequency [28]. Noise from cargo ship was chosen because it is one of the largest contributors to ocean noise pollution. The two other most significant sources are oil and gas exploration and military sonar. The cargo ship contribution is greatest in coastal areas which are hotspots of biodiversity.

## Materials and methods

### Animals and experimental design

Experiments were performed at Arcachon Marine Station, on the French Atlantic coast, from October to November 2016. A total of 120 18-month-old diploid Pacific oysters *M*. *gigas* (70–75 mm) were purchased from a local oyster farmer in Arcachon Bay and maintained in large tanks (200 L) filled with running unfiltered seawater pumped directly from the bay. Arcachon Bay experiences low-density marine traffic composed of recreational and small fishing ships (most of them < 10 m).

The experiment was conducted in an isolated room to limit external influences on the oyster behavior. Care was taken to reduce human activity and noise disturbance. Four identical polyvinylchloride tanks (78 x 39 x 19 cm) were used, with animals being acclimated to the experimental conditions for a week. The tanks were supplied with unfiltered sea water with a controlled renewal rate of 130 mL/min using a flowmeter (Cole-Parmer, USA). Water flow was piped underwater, thereby minimizing the noisy disruption of water-surface and background noise. Before and during the test exposures, oysters received 0.4 g of dehydrated algae (ReefMist Planktonic, Aquarium System) for feed once a day before the lights were extinguished to optimize food uptake by the oysters. Animals were subjected to a light cycle of L:D 13:11 in the absence of a tidal cycle, and the water was air-equilibrated by slow bubbling air stones set in airlifts to homogenize the water volume and limit unnecessary noise. Physical parameters were monitored during all experiments and maintained at 14–15°C; pH, 7.8–8.0; and salinity, 32-34/1000. Among the four tanks, two (replicates) were dedicated to the cadmium and cargo ship noise test; two (replicates), to the cadmium only test. Eight animals per tank were equipped with electrodes for behavioral measurements (8 x 2 replicates = 16 oysters per condition; see below).

**Fig 1:**
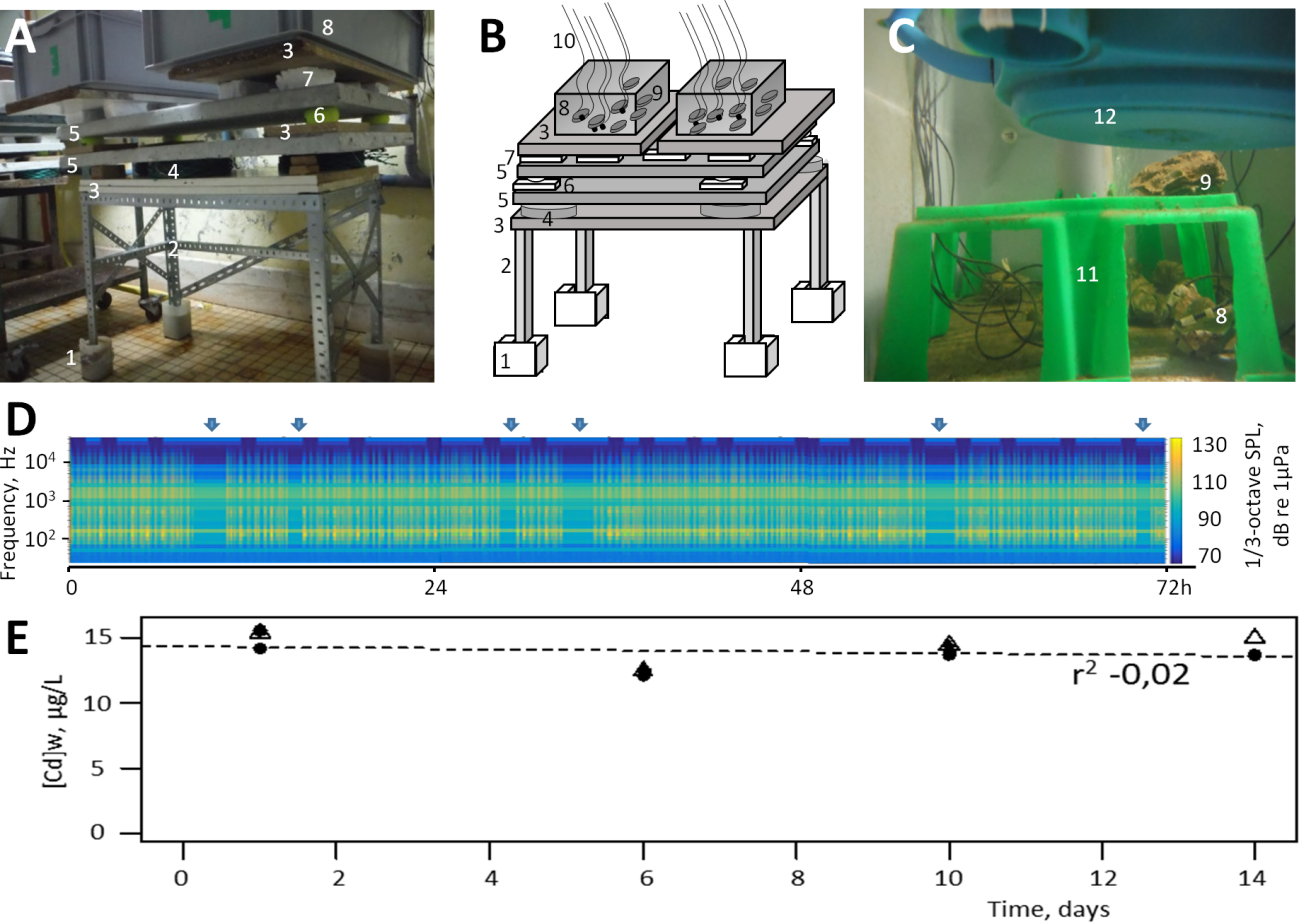
Experimental setup and exposure characteristics. A, antivibration bench with 2 replicate tanks on it; B, complete scheme of a unit; C, inside view of an experimental tank. 1, sand; 2, frame angle structure; 3, chipboard panel; 4, air chambers; 5, concrete slab; 6, tennis ball; 7, pieces of thermal insulation; 8, oysters equipped for behavioral recordings; 9, oysters for tissue sampling; 10, electrode cables; 11, hemstitched support; 12, loudspeaker. D, 72 h of sound recording. Each vertical bar is the noise from one passing cargo ship. Black bands represent periodic absence of cargo ship noise. E: the change of cadmium concentration in the 4 tanks as a function of time. There were 2 antivibration benches and 2 replicate tanks per bench.

Due to its acoustical mismatch, the air-water interface causes the reflection of a large part of the acoustical energy [29]. Therefore, the main conduct of unwanted sound transmission into the tanks was through the base. To limit ground vibrations, tanks were installed on an antivibration bench. The system is composed of 12 layers of different materials (Fig 1A and 1B) composed of a plastic box, sand, chipboard, inner tubes, concrete, tennis balls, polystyrene boards, and PVC) to absorb extraneous vibrations and to prevent parasite disturbance of the oysters’ ventilatory activities [30]. Two suspended loudspeakers were installed face down in the two replicate tanks dedicated to the Cd and noise exposure. They were positioned at the center and 12 cm from the bottom (Fig 1A).

Exposure protocol. T_0_ was taken as the first day of exposure to cadmium, and T_0_-T_14_ was the exposure period to cadmium with or without the cargo ship noise. After an acclimation period of 6 days to the experimental tanks (from T_-11_ to T_-5_), oysters placed in the two tanks equipped with loudspeakers were exposed to cargo ship noise (from T_-5_ to T_14_; Fig 1D) while the two reference tanks (no noise) remained in a reference condition (T_-5_ to T_0_). During the test, the loudspeakers were switched off every day from 09 h 00 to 11 h 00 during animal sampling (Fig 1C).

From T_0_ to T_14_, metal contamination was initiated in all four tanks with the addition of CdCl_2_ from a stock solution (Titrisol®, Merck). The average concentrations in tanks 1 and 2 with cargo ship noise were respectively 13.91 ± 0.72 μg∙L^-1^ and 13.78 ± 0.71 μg∙L^-1^. It was respectively 14.32 ± 0.64 μg∙L^-1^ and 13.59 ± 0.39 μg∙L^-1^ for tanks 3 and 4 without cargo ship noise (means ± 1 SE; Fig 1E). The expected maximum free Cd concentration was < 3–4% of the total Cd, only taking into account the inorganic complex (MINEQL+, V5) that is ≤ 0.5 μg∙L^-1^.

Five oysters without HFNI electrodes were randomly sampled at T_0_, T_3_, and T_7_, and seven were sampled at T_14_ in each tank to give n = 10 or 14 per experimental condition and exposure period. The gills and digestive gland were immediately sampled and divided into two parts: one for measuring the concentration of Cd in the tissues and one for cDNA synthesis and quantitative PCR. The samples were stored at -20°C for metal determination and fixed as explained below for genic analysis.

### Sound production and recordings

Playback tracks were created from original recordings made at Santander Harbor (Spain). During these recordings, the hydrophone was positioned at a depth of 2 m, with cargo ships passing at a distance of approximately 500 m. Due to port regulations, the ships were estimated to be sailing at a speed ≤ 9 knots. Weather conditions were calm (wind force 3 on the Beaufort scale). A noise generated by one passing ship was randomly picked. The selected noise of 12-min duration was modified using a sound program interface (Cool Edit, version 2.0, Syntrillium Software Corporation, USA) to obtain two records with two different amplitudes and with frequencies from 20–20,000 Hz: one passing ship had a maximum amplitude of 150 dBrms re 1 μPa, and the other had a maximum amplitude of 138 dBrms re 1 μPa. The noise record is provided as supporting information (S1 File).

Three different playbacks of 1-hour duration each were created. The tracks contained, respectively, three, four, and five passing ships alternating large and small amplitudes with silent periods in between for a total of 92 noise exposure periods per day (Fig 1D). This is a value representative of active ports (https://www.marinetraffic.com/). The three playbacks were randomly dispersed over a period of 3 days, and the final track was looped for the experimental exposure. Sound was played back as a wave file through a loudspeaker (model: US-0130, Rondson Public Address, France) using an amplifier (AM60A, Rondson, France) and Cool Edit as an audio interface.

Acoustic conditions were recorded using a broadband hydrophone with an internal buffer amplifier (model: H2a-XLR, sensitivity -180 dB re 1 V/μPa, useful range: 10 Hz to 100 kHz, Aquarian product) and an Edirol recorder (H4n Handy, Zoom Corporation, Japan) that was level-calibrated using a pure sine wave signal with a measured voltage recorded in line on an oscilloscope. Recordings were made (6 cm) above the tank floor and 12 cm from the loudspeaker.

As reported by Rogers et al. [31], measuring particle velocity in small tanks is a challenge. In this experiment, we measured acceleration directly on *in situ* oysters by attaching a Dytran accelerometer (3217A-10) to the shell of a test specimen [20]. Its certified sensitivity varied from 100.38 mV/g at 20 Hz to 96.96 mV/g at 1000 Hz, and we used it with a Dytran amplifier (M28 IEPE, gain x 1) and a 100x laboratory-made amplifier. The accelerations were measured at different positions in the tanks (Fig 1C) and ranged from 0.32–0.43 m·s^-2^ at 150 dBrms re 1 μPa and from 0.06–0.08 m·s^-2^ at 138 dBrms re 1 μPa.

### Oyster behavior recording

As explained by Andrade et al. [21], the recording apparatus consisted of lightweight electromagnets (56 mg) glued to each valve and connected by flexible wires to a laboratory-made valvometer. The device measures current which varies according to the distance between the electrodes. For this experiment, the sampling frequency was 3.3 Hz, and according to that frequency, the opening status of each oyster was measured every 4.8 sec.

Data were transmitted via an acquisition card (Ni-USB-6009, National instrument, Austin, TX, USA) and recorded by a computer using a laboratory-made script in Labview (National instrument). Data were also processed automatically using routines and published online on the professional pages of the MolluSCAN eye website https://molluscan-eye.epoc.u-bordeaux.fr/index.php?rubrique=accueil&lang=en&site=EYRAC.

In bivalves, a change in the valve behavior could indicate a disturbance in the surrounding environment. The main interest of such a device was to collect online different parameters without interfering with the normal behavior of the bivalves: valve closure duration, valve partial closure and growth rate. The valve closure duration is based on the measurement of the time spent by the oyster closed, which can be calculated hourly or for the entire day. Measurements are based on the use of several thresholds. Thresholds are calculated while considering the minimum and maximum opening amplitude for each oyster over 24 hours. Different thresholds could be chosen (5%, 30%, 55%, and 80%) depending on the studied parameter. An actogram was used to represent the temporal distribution of the valve opening duration activity for the oyster group. Each line in the actogram represents the group's activity in 24 hours. A daily mean activity was calculated for the group. For each time slot, the mean hourly activity of the group was compared with the calculated daily mean activity. When the activity was below the average, it is represented with a white bar code, whereas values above the average are represented with a black bar code. The actogram can be calculated at different thresholds. In this study, the 5% threshold was chosen because it allows differentiating between opened and closed.

The valve closure duration is the percentage of the ratio between the maximum and minimum opening amplitude of the oyster for the whole day. The hourly valve closure duration could also be expressed as a percentage at different thresholds. A duration of 100% corresponds to an oyster being continuously opened for 1 hour, and 0% corresponds to an oyster being closed for the entire hour. Intermediate values are possible.

Valve partial closure is calculated from the sum of the detected partial closures for a given time for each animal at different thresholds. In the present experiment, the 80% thresholds were used for partial-closure calculation, as this was appropriate.

Growth rate can be estimated using valvometry. As calcification in bivalves occurs in the mantle cavity, when daily growth layers are deposited, the minimum distance between the electrodes glued to the shells increases, thereby providing a good index of growth. By plotting these values of minimal daily valve opening as a function of time, one obtains growth curves [32]. For each condition, no significant difference was observed between the duplicates, and the data were pooled together.

### Cadmium contamination and determination

Cadmium concentration in the water was maintained and monitored during all experiments. Cadmium was pumped at a rate of 0.24 mL/min and injected in the inflow pipe using a peristaltic pump (Gilson, France). Water aliquots, filtered at 0.45 μm, were collected daily in the experimental tanks to monitor water contamination. After acidification and dilution of the aliquots, the cadmium contamination was measured with ICP (Inductive Coupled Plasma. Model: 700 series ICP-OES, Agilent Technologies). Oyster samples (gills and digestive gland) were dried for 48 h at 45°C and then digested with nitric acid (Nitric Acid 65%, Carlo Erba Reagents, France) in a Teflon tube at 100°C for 3 h. The resulting solutions were diluted with ultrapure water (Purite Select Purification System), and cadmium concentrations were determined by atomic absorption spectrometry with Zeeman correction (Model: 240Z AA, Agilent Technologies). To avoid interference, samples were mixed before atomization with a 1-mL mixture of 50% Pd + 50% Mg (NO_3_)_2_. The method was simultaneously checked for each sample series against certified biological reference materials (Dolt-5, National Research Council Canada). Values were consistently within certified ranges. Metal concentrations were expressed as (μg/g) dry weight tissue. No significant difference was observed between the oysters in replicate tanks under the same experimental conditions. Thus, data were pooled together.

### cDNA synthesis and quantitative PCR

Gill samples of oysters were individually processed for RNA extraction using TRI Reagent® (Invitrogen, Carlsbad, CA, USA) according to the manufacturer’s instructions. Total RNA quality and quantity were estimated by spectrophotometer (Epoch, Biotek®), and reverse transcription was realized with 4 μg total RNA, oligo dT17, and Moloney Murine Leukemia Virus (M-MLV) reverse transcriptase (Promega, Madison, WI, USA).

Nineteen genes were chosen to encode the proteins involved in the various metabolic pathways. For each candidate, specific primers were designed from the sequence of *M*. *gigas* available in the National Center for Biotechnology Information (NCBI) database or selected from the literature (Table 1). The PCR efficiency of each primer pair was realized by determining the slope of the standard curve obtained from the serial dilution analysis of cDNA. Real-Time quantitative Polymerase Chain Reactions (RT-qPCR) were performed in an Mx3000P (Stratagene®) with GoTaq qPCR Master Mix (Promega®, Madison, WI, USA) and a concentration of 100 nM for each primer according to the manufacturer’s instructions. The amplification program starts with 3 min at 95°C to activate the DNA polymerase, followed by 50 cycles of 10 sec at 95°C and 30 sec at 60°C. Reaction specificities were confirmed by a melting curve (from 65°C to 95°C). Five potential housekeeping gene (*12s*, *ef1α*, *rpl7*, *gapdh*, *actb*) was tested using BestKeeper, geNorm and Normfinder and *12s* gene was sectioned. The expression of transcripts levels in each oyster was quantified by the 2^-ΔΔCt^ method described by Livak and Schmittgen [33].

**Table 1:** Primers used for the RT-qPCR assay.

Genes		Functions	Primer sequences	Accession numbers	References
*clec1*	C-type lectin-1	Feeding selection	F- GTGAGACCGGAACTGGATTAG	AB308130.1	This study
			R- CGTTGAAGACCTGGAGCAT		
*drd2*	Dopamine receptor D2	Signalisation	F- GTGAGCGTGATTCTCTGGTAG	JH817851.1	This study
			R- CGAGCGACCTTAGTCAGTTT		
*dld*	Dihydrolipoyl dehydrogenase	Krebs cycle	F- GGAGCTTCCTGTGAAGACATAG	XM_020071857.1	This study
			R- CTGCATAGGCTGCCAAGTTA		
*cs*	Citrate synthase		F- ACAGTCCTGTTCGGAGTTTC	JH816386.1	This study
			R- ACGACTTGGGACGTTCAAT		
*mdh*	Malate dehydrogenase		F- GCTGGAACTGAGGTTGTAGAG	XM_011435972.2	This study
			R- GCACCATTCAAGGCATCAAG		
*idh2*	Isocitrate dehydrogenase [NADP] mitochondrial		F- CCGACGGAAAGACTGTCG	AY551096.1	[34]
			R- CTGGCTACCGGGTTTGTG		
*idh3a*	Isocitrate dehydrogenase [NAD] subunit α		F- GTCATGATGCTCCGCTACAT	JH816185.1	This study
			R- CAGTTAGCACCCTTCCTTCTC		
*tnt*	Titin	Muscular activity	F- TTTGGTACTGCGGAGTTCTG	JH817834.1	This study
			R- CTCCGGCAAATGGTGAGTAT		
*twt*	Twitchin		F- CCGAAACTCCTCCAACTCAA	XM_020069067.1	This study
			R- TCCTCAGCAGCTATCCTATCA		
*cox1*	Cytochrome c oxidase subunit I	Mitochondrial metabolism	F- GTGCCAACTGGTATTAAGGTGT	AB033687	[35]
			R- ACACCGCACCCATTGAT		
*sod2*	Manganese superoxide dismutase	Oxidative stress	F- ACAAAGTCAATCAGTGCCCT	EU420128	[35]
			R- CCATTGCCTCTGCCAGT		
*sod1*	Copper/Zinc superoxide dismutase		F- CCAGAGGATCACGAGAGGC	AJ496219	[36]
			R- GCGTTTCCGGTCGTCTT		
*cat*	Catalase		F- GTCGTGCCCCTTTACAACC	EF687775.1	[35]
			R- CGCCCGTCCGAAGTTT		
*gpx*	Selenium-dependent glutathione peroxidase		F- ATCGAACGCTGCACCA	EF692639	[35]
			R- AGCTCCGTCGCATTGT		
*mt2*	Metallothionein isoform 2	Detoxification	F- TCCGGATGTGGCTGCAAAGTCAAG	AJ297818	[37]
			R- GGTCCTTTGTTACACGCACTCATTT		
*acac*	Acetyl-coA carboxylase	Lipogenesis	F- GGGCACCGTTAATGCCTAC	XM_020073360	[38]
			R- TGACTCGGGGTCATGTGTT		
*tgl*	Triacylglycerol lipase	Lipolysis	F- ACACCCACCGTGCTTT	EE677866	[38]
			R- GTTTGTCTTGAGATCCTTGATTATCAG		
*p53*	p53 gene	Cell cycle / apoptosis	F- CCCTCAAAGCAGTCCCCA	AM236465.2	[35]
			R- TGTAGCGATCCACCTGATT		
*bax*	BCL2 associated X	Apoptosis regulator	F- CCCTCAGGTGTGACCCG	XM_011426179	[38]
			R- TGCAACGTAAAGCTCTGCC		
*12s*	12S ribosomal RNA	Housekeeping genes	F- CTCAGTCTTGCGGGAGG	EF484875	[35]
			R- GGTTATGCGGAACCGCC		
*ef1α*	Elongation factor 1 α		F- AGAATGGATATGCGCCTGT	AB122066.1	[38]
			R- GCCACGGTCTGCCTCA		
*rpl7*	Ribosomal protein L7		F- ACACCTCGGACGCTTT	AJ557884.1	[38]
			R- GCTGTCTTCACGCAGGC		
*gadph*	Glyceraldehyde 3-phosphate dehydrogenase		F- CGTACCAGTTCCAGATGTTTCC	CAD67717	[39]
			R- GCCTTGATGGCTGCCTTAATA		
*actb*	β-actin		F- AGTACCCCATTGAACACGG	AF026063	[35]
			R- TGGCGGGAGCGTTGAA		

F and R designate forward and reverse primers respectively.

### Statistics

Comparisons between variables were investigated using analysis of variance (ANOVA) after checking for assumptions of normality and homoscedasticity of error term. For significant differences, the Tukey’s Honest Significant Difference (Tukey’s HSD) was used for all pairwise comparisons. When assumptions were not met, the Kruskal-Wallis test was applied, and the Dunn test was considered for all pairwise comparisons. Differences between distributions were assessed using a two-sample Kilmogorov-Smirnov test, and differences between variances were assessed using a Levene test. For all statistical results, a probability of p < 0.05 was considered significant. Data were computed and analyzed using R software [40]. All data are provided as supporting information (S1 Database).

## Results

### Cadmium accumulation

**Fig 2:**
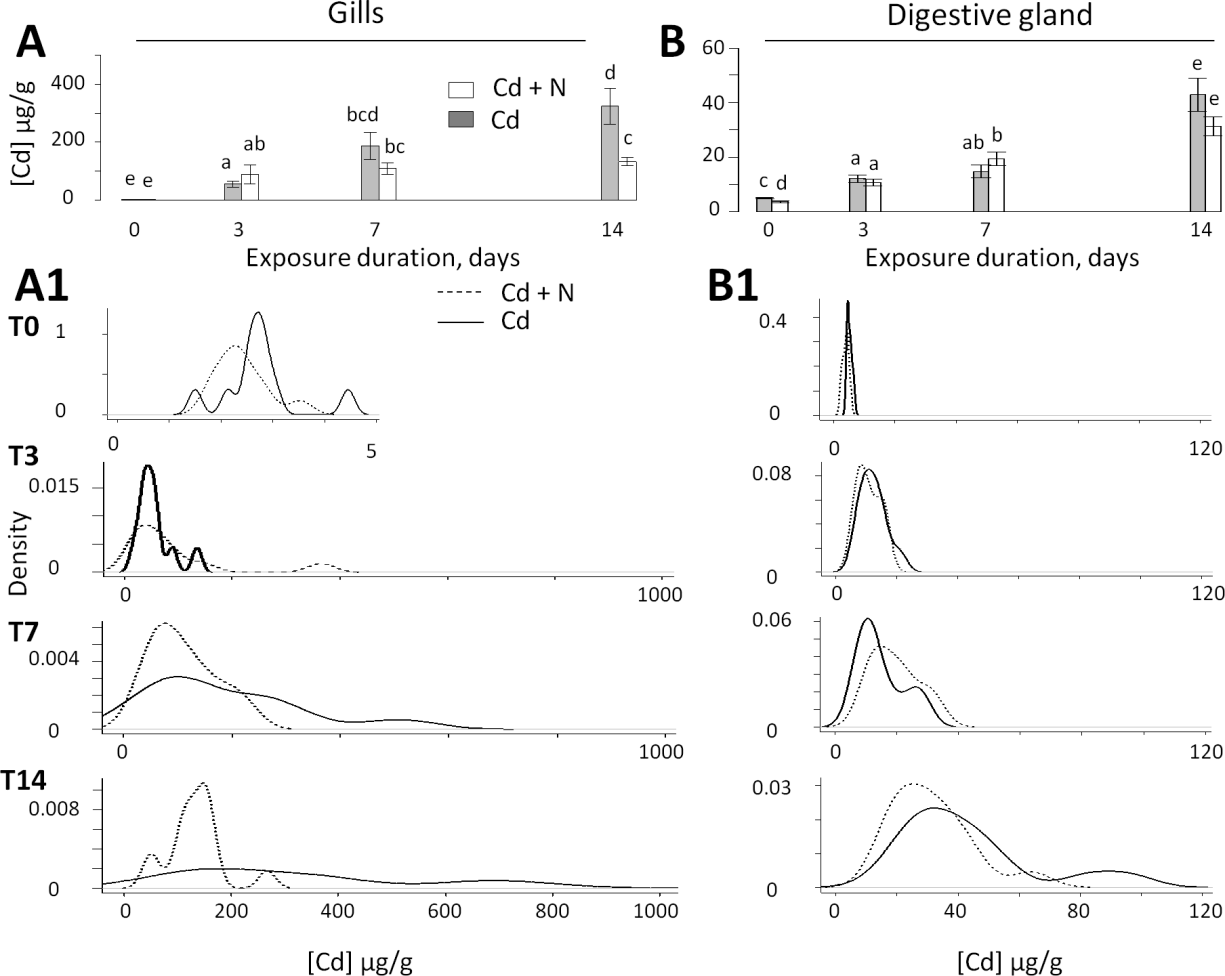
**The change of cadmium concentration in the gills (A, A1) and digestive glands (B, B1) in oysters *M*. *gigas*.** A: mean values ± 1 SE as a function of time in the gills; B, in the digestive glands. A1 and B1, Distribution of probability densities at day 0 (T0), day 3 (T3), day 7 (T7) and day 14 (T14). In the presence of cargo ship noise, the distributions were skewed to the lower values at T_3_, T_7_, and T_14_ in the gills and T_14_ in the digestive glands.

Cadmium concentrations in the gills ([Cd]g) and the digestive glands ([Cd]dg) of the oysters exposed to Cd with or without cargo ship noise are reported in Fig 2. After 14 days of Cd exposure, the gills of oysters exposed to cargo ship noise accumulated 2.5 times less than the oysters without ship noise ([Cd]g = 132. 6 ± 14.2 μg∙g^-1^ dry w in noise-exposed oysters versus 323.2 ± 61.4 μg∙g^-1^ dry w in non-exposed oysters; p = 0.009). From T_3_ to T_14_, the [Cd]g increased by 480% in the absence of ship noise and only 48% in the presence of cargo ship noise. By contrast, in the digestive gland, which accumulated 4–8 times less Cd than the gills, no significant difference was observed between the noise conditions regardless of the exposure duration (p = 0.137 at T_14_).

The distributions of cadmium accumulation in the two oyster groups all along the exposure period are presented in Fig 2A1 and 2B1. Fig 2A1 shows that the difference between the oysters exposed and not exposed to cargo noise was mainly explained by the absence of the most contaminated gill samples in the cargo-noise-exposed condition. After 14 days, the distributions in the two conditions became significantly different (p = 0.0049), but at T_3_ and T_7_, the higher contaminations levels were already associated with the absence of cargo ship noise. In the digestive glands, the cadmium level distribution in the absence of ship noise was not different regardless of the length of exposure (p = 0.994 at T_3_, 0.4175 at T_7_, and 0.635 at T_14_; Fig 2B1). To gain more insight into the mechanism leading to this difference in bioaccumulation, we then turned to a comparative analysis of the oyster behavior in the presence and absence of cargo ship noise.

### Animal behavior

**Fig 3:**
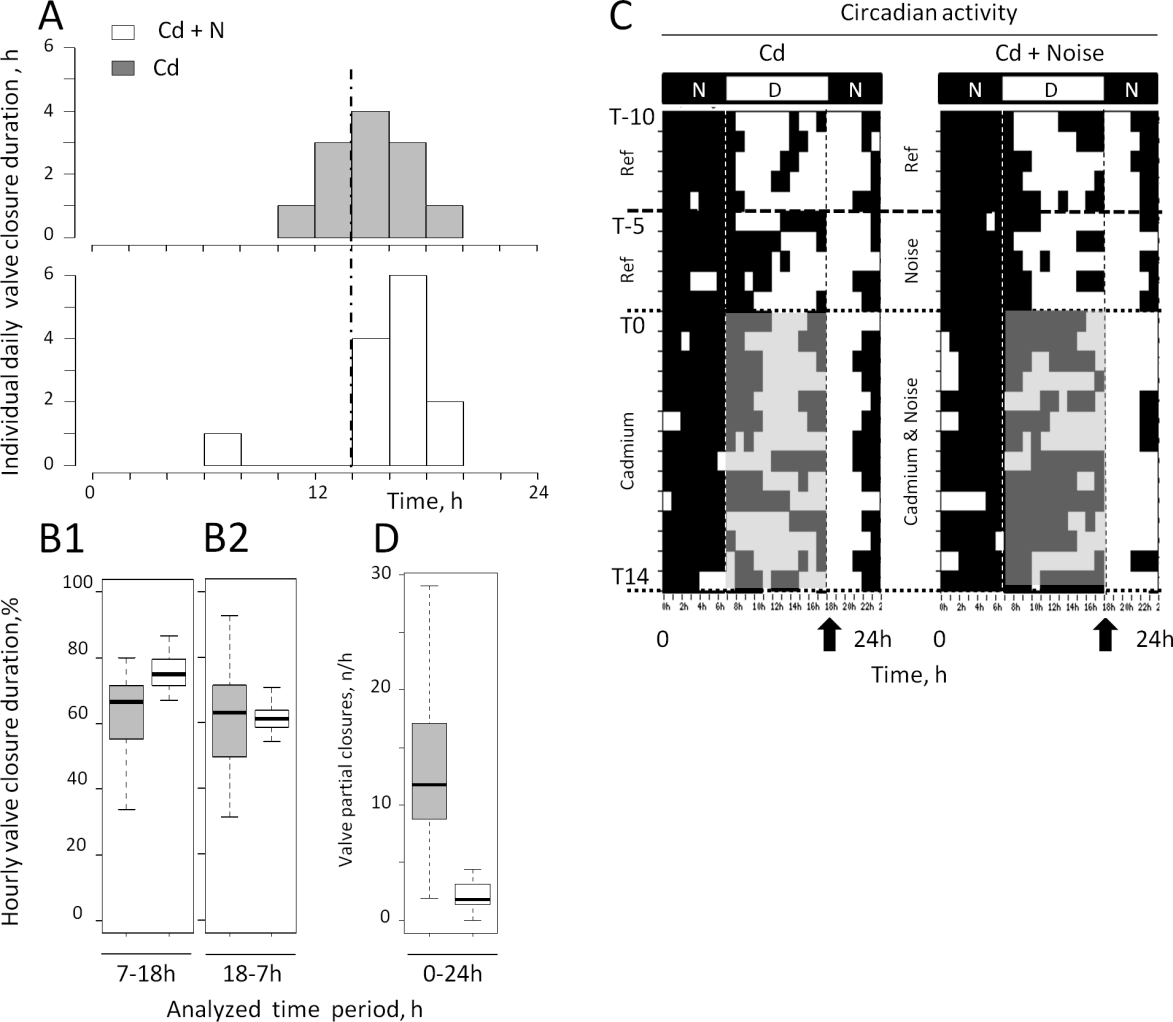
Behavioral changes in the presence of cargo ship noise in oysters *M*. *gigas* exposed to Cd. A, Distribution of individual valve closure durations. B1, Description by quartiles of the hourly valve closure durations during the light (7-18h) and B2, dark (18-7h) periods in oysters exposed to Cd plus cargo ship noise (white) or Cd alone (gray). D, number of partial valve closures per hour. A, B1, B2, and D were calculated for the whole 14-day-exposure period; bold lines, medians. With one exception at the bottom of Fig 1A, the variability was systematically lower in the presence of cargo ship noise. C, Valve opening—closing actograms, each line represents 1 day. The white and black sections of each line represent hourly behavioral values above and below the mean behavior of the day, respectively. In the presence of cargo ship noise, the oysters are closed more during the day (light) period, gray area. The black arrows show the daily timing of the food supply, just prior the opening behavior associated with the automatic light shut-off. N = 13 oysters exposed to Cd and cargo ship noise and 12 oysters exposed to Cd alone.

During the first hours of noise exposures at T_-6_ and T_-5_, a transient valve closure, as reported in Charifi et al. [20], was observable in 100% of the animals. The response was present in 60% of the individuals after 2 days and vanished after 4 days. Fig 3A presents the distribution of the mean daily valve closure durations for oysters under the two conditions for the entire 14-day exposure period to Cd. In the presence of cargo ship noise, a shift occurred toward higher values in the most frequently observed mean hourly valve closure durations. No significant difference was observed between the mean values (p = 0.09764) due to a single outlier (p = 0.0001) in the group exposed to ship noise. In the absence of this outlier, the difference became significant (p = 0.02982), which illustrates the small difference between conditions. We then compared the diurnal and nocturnal periods (Fig 3B1 and 3B2). During the diurnal period (7–18 h, active period), an increase was observed in the valve closure duration in the oysters exposed to ship noise (Fig 3B1; p = 0.029) but not during the nocturnal period from 18–7 h (Fig 3B2; p = 0.857). This result is also illustrated in Fig 3C, which presents the hourly circadian activity under both conditions: compare the two shaded areas comprised between T_0_ and T_14_ during the diurnal period. Finally, the depressant action of noise was also characterized by a drastic decrease in the valve activity measured, as shown by a decrease in the number of valve movements crossing the 80% line of maximum opening value under the Cd plus ship noise condition (Fig 3D). In the absence of ship noise, the average number of valve partial-closures per hour was approximately six times higher, and the variability was more important than in the oysters exposed to the Cd plus ship noise (min = 0.00∙h^-1^; median = 1.80∙h^-1^; max = 4.47∙h^-1^ under cargo-ship noise and min = 1.94∙h^-1^; median = 11.78∙h^-1^; max = 29.00∙h^-1^ without cargo-ship noise, p = 0.0003).

We then studied some consequences of the phenomenon at the genic level in the gills, as the gills exhibited a significant change in contamination, before switching to an analysis at a more global and integrative level for the comparison of growth rates.

### Gene expression in the gills

**Fig 4:**
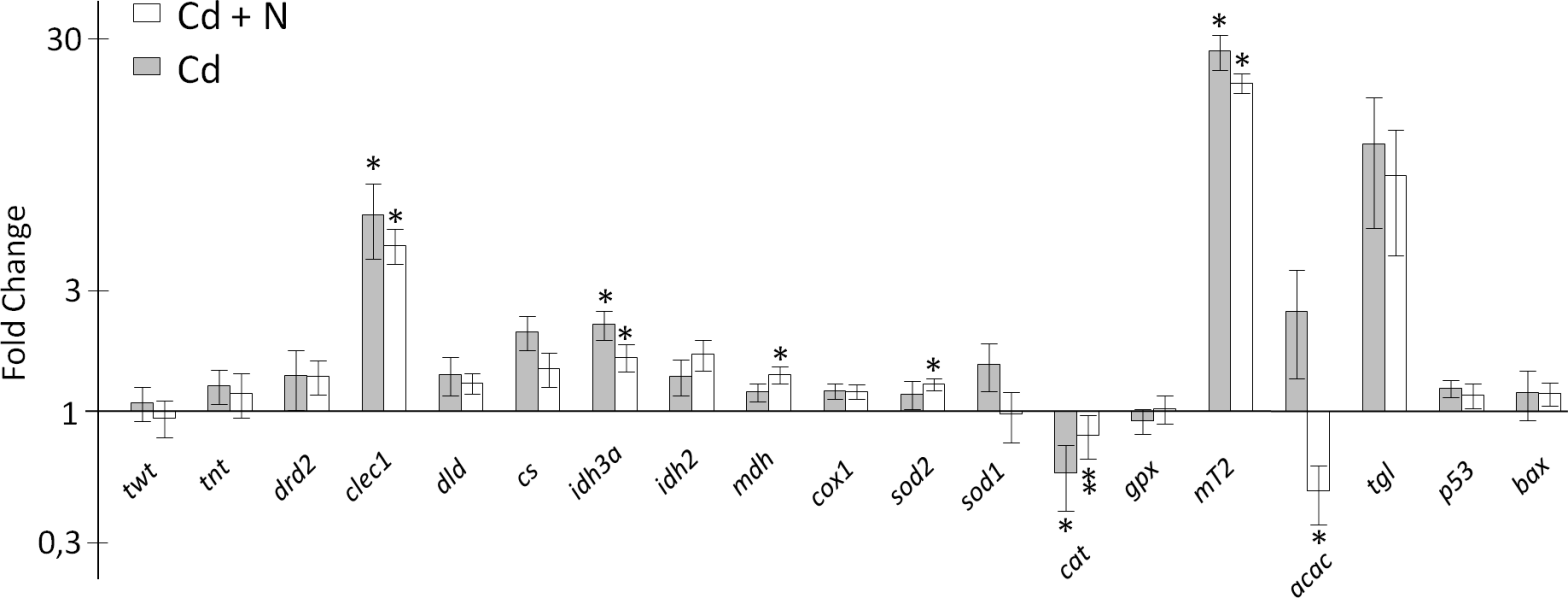
Transcriptional variations of selected genes in the gills of oysters *M*. *gigas* exposed to Cd plus cargo ship noise or Cd alone for 14 days. * and ** denote significant differences between the condition and the control and between experimental conditions, respectively.

**Fig 5:**
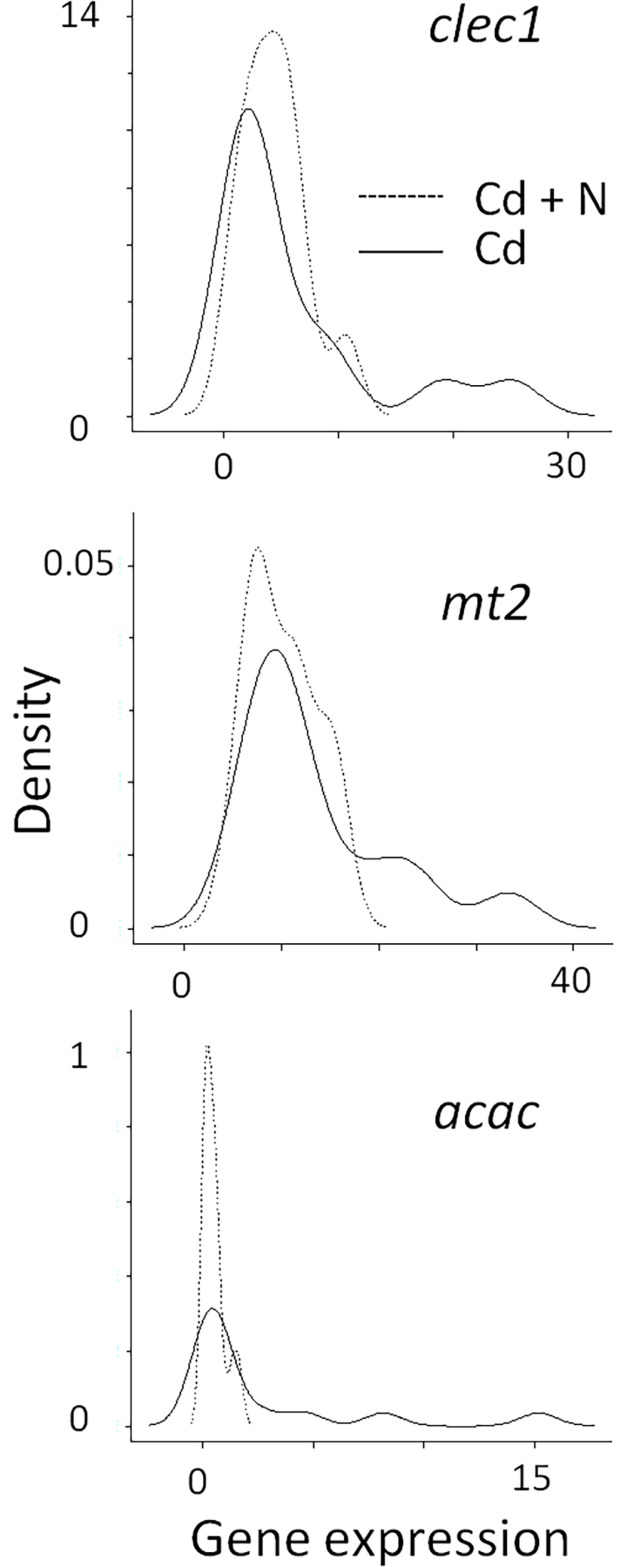
Distribution of the probability densities of gene expression in three genes exhibiting a different variance at day 14. In the presence of cargo ship noise (Cd + N), the distributions were skewed to the lower values in comparison to the distribution in the absence of the ship noise (Cd).

In the absence of cargo ship noise and after 14 days of exposure to Cd alone, 4 of the 19 genes were over- or under-expressed compared to the reference at T_0_ (*clec1*, *idh3a*, *mt2* and *cat*; Fig 4). In the presence of cargo ship noise and Cd, the expression of 7 of the 19 genes was modified (*clec1*, *idh3a*, *mdh*, *sod2*, *cat*, *mt2*, *acac*). Only one gene was differentially expressed between both conditions: *cat* (p = 0.045). As expected, the *mt2* gene, which is a hallmark of cadmium contamination, exhibited large induction factors of 20x (with ship noise) and 27x (without ship noise). The *clec1* gene was also largely overexpressed (4.6-6x). In addition to differences between mean or median values, the variability in the gene responses was also studied to learn if the presence of cargo ship noise influenced it in a manner consistent with that observed at bioaccumulation and behavioral levels (Fig 2A1; Fig 3B1 and 3B2 and 3D). For each gene, we researched if the expression increased when [Cd]g was larger. Based on the Pearson correlation, the results in Table 2 show that it is only in the absence of ship noise for *dld*, *cs*, *idh3a*, *sod1*, *gpx* and *bax* that significant correlations are observed. The distributions of expression with and without cargo ship noise were either unchanged or skewed to the lowest values in the presence of the cargo ship noise. Levene’s test demonstrated that the homogeneity of variance was different between the ship noise conditions for *mt2* (p = 0.0322), *acac* (p = 0.0036), and *clec1* (p = 0.0221). The distributions of expression were smaller under the condition cadmium plus cargo ship noise (Fig 5).

**Table 2:** Pearson correlation of [Cd]g and gene expression in Cd and Cd+N conditions.

Cd	*twt*	*tnt*	*drd2*	*clec1*	*dld*	*cs*	*idh3a*	*idh2*	*mdh*	*cox1*
	0.02	-0.21	-0.34	-0.36	0.82	0.58	0.74	0.44	0.29	0.39
	NS	NS	NS	NS	***	*	**	NS	NS	NS
	*sod2*	*sod1*	*cat*	*gpx*	*mt2*	*acac*	*tgl*	*p53*	*bax*	
	0.45	0.6	0.47	0.62	0.14	0.31	0.42	-0.07	0.71	
	NS	*	NS	*	NS	NS	NS	NS	**	
Cd+N	*twt*	*tnt*	*drd2*	*clec1*	*dld*	*cs*	*idh3a*	*idh2*	*mdh*	*cox1*
	-0.18	0.07	0.42	0.2	0.4	-0.08	0.12	0.22	-0.04	0.09
	NS	NS	NS	NS	NS	NS	NS	NS	NS	NS
	*sod2*	*sod1*	*cat*	*gpx*	*mt2*	*acac*	*tgl*	*p53*	*bax*	
	0.29	-0.33	0.16	-0.1	0.35	0.26	-0.47	0.2	0.08	
	NS	NS	NS	NS	NS	NS	NS	NS	NS	

NS, Not statistically significant

(*), p<0.05

(**), p<0.01

(***), p<0.001.

### Growth rate

**Fig 6:**
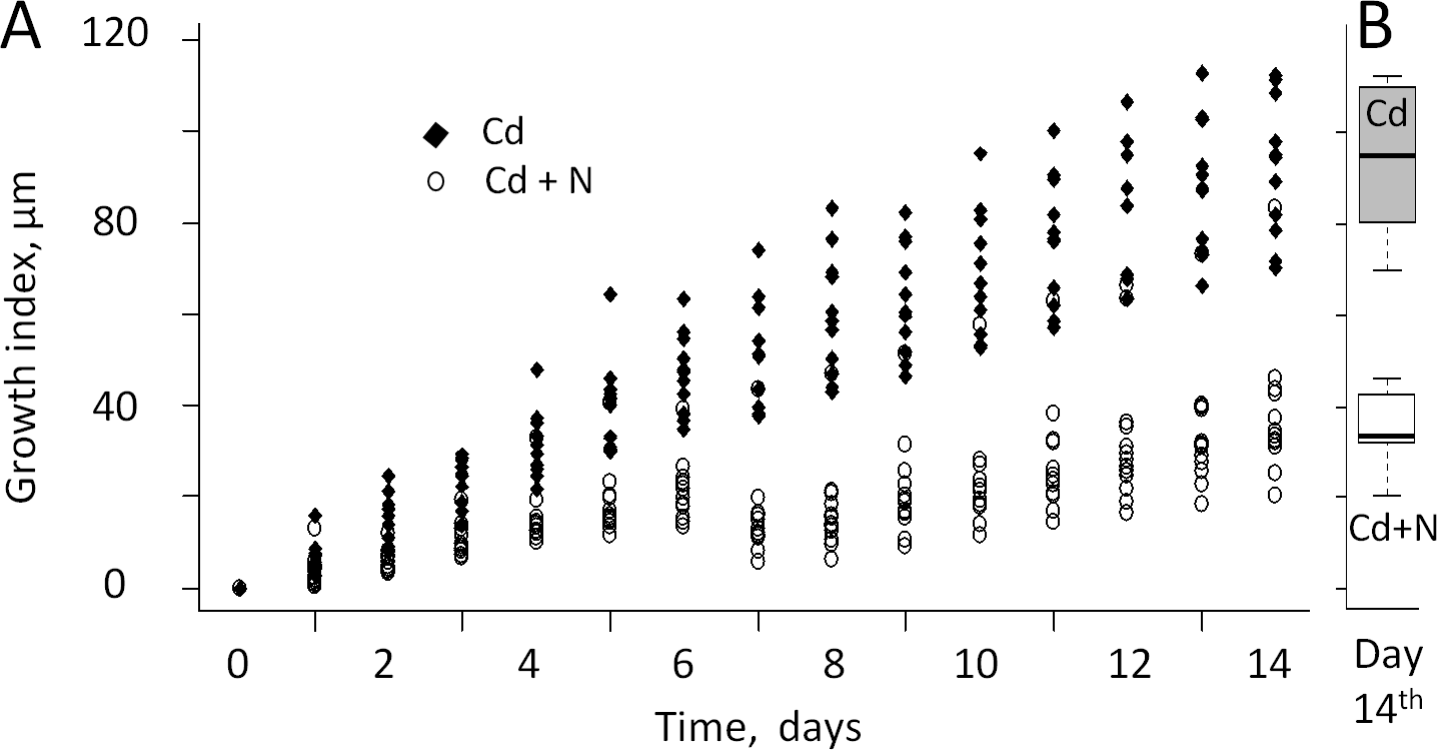
Growth rate index in oysters *M*. *gigas* in the presence of cadmium with (Cd + N) or without (Cd) cargo ship noise. A, daily change of growth rate index. B, description by quartiles of the difference between growth index at day 14. The growth rate is smaller in presence of cargo ship noise. N = 13 oysters exposed to Cd and cargo ship noise and 12 oysters exposed to Cd alone.

HFNI valvometry allows information to be recorded on the daily growth rates under various experimental and environmental conditions without the animals being handled. We therefore compared the oysters shell growth rates in the presence and absence of cargo ship noise. Fig 6A and 6B shows that the growth rate was lower in oysters exposed to frequent ship noise compared with the control group without ship noise (p = 0.00001). At T_14_, the mean size gain at the HFNI electrode level (growth index) reached only 37.9 ± 4.2 μm in the presence of cargo ship noise when it was 99.6 ± 8.8 μm in the absence of ship noise (p = 0.000004; Fig 6B). Moreover, starting at T_6_ a growth arrest was observed in the presence of cargo ship noise, as the growth indexes did not vary from T_6_ to T_10_ (p = 0.542; Fig 6A). No similar trend was observed in the non-noise group.

## Discussion

The influence of chronic exposure to cargo ship noise (92 ship passages/day) on metal contamination was studied in oysters during a 14-day exposure period. We compared two situations with similar concentrations of [Cd]w and food supply in the presence or absence of ship noise. We analyzed the accumulation of Cd in the gills and digestive glands, the transcriptional response of selected genes in gills, the valve behaviors, and the growth rates. Based on previous works, cadmium was taken as both a toxicant and an indirect marker of ventilatory activity. The whole data set illustrated the power of noise as a physiological drive and suggested a depressant effect characterized by a decrease of valve opening duration, valve activity, ventilatory activity and growth rate. The associated ecotoxicological impact was a decreased Cd bioaccumulation in the gills and a modulation of transcriptional expression of some genes. Indeed, *mt2*, *clec1*, and *acac* exhibited the largest changes. Finally, an analysis of variance for the whole data set (Cd concentrations in gills and digestive gland, valve closure durations, number of partial valve closure, gene, and growth rate) showed that variance frequently decreased in the presence of cargo ship noise with most individual data skewed to the lower range.

### Impact of noise on ventilatory activity

Few studies have reported the effect of anthropogenic noise on the ventilatory activity in marine animals. Bruintjes et al. [41] recorded transient hyperventilation in juvenile European eels (*Anguilla anguilla*) and the juvenile European seabass (*Dicentrarchus labrax*) when exposed to a short noise stimulus (eel: ship noise for 2 min at 139.3–141.2 dBrms re 1 μPa; seabass, pile driving for 2 min, 165–167 dB re 1 μPa at peak level). The hyperventilation rapidly dissipated (within 2 min) after exposure in the seabass, and the recovery was partial in the European eels. In crustaceans, Wale et al. [42] reported that crabs *Carcinus maenas* exposed to a single, ship-noise playback every 2 days over 15 days (148–150 dBrms re 1 μPa, duration 7.5 min) exhibited a reproducible increase in oxygen consumption without acclimation with time. Radford et al. [43] conducted an experiment over 12 weeks. They showed that naive fish hyperventilated when exposed to a single impulsive noise but not when exposed to repetitive stimulation. It fits well with the present study as oysters exhibited a transient decrease in their valve opening amplitudes—similar to that described by Charifi et al. [20]–only at the beginning of the test period. It faded over time to vanish after 4 days. The ventilation, defined as the volume of water ventilated per unit of time, was not directly measured in this report because it required handling, and we did not want to interfere with the animal’s resting behavior. Instead, we used the Cd bioaccumulation as a proxy because in water breathers that were maintained at a constant [Cd]w, an increase in ventilatory activity has been demonstrated to increase the Cd accumulation and vice versa (see Introduction). In the present experiment, possibly the oysters exhibited a transient hyperventilation at the onset of the cargo ship noise exposure but the bulk of evidence shows that they ventilated less during most of the 14-day exposure period (see Fig 3).

Oysters unexposed to ship noise presented a larger interindividual distribution of [Cd]g, with more contaminated animals. As reported by Pan et al. [44] in the scallop *Chlamys nobilis*, such an intraspecific variability is routinely observed in metal bioaccumulation for individuals of the same size and collected at the same location and period. This is also similar to the PSP toxin accumulation observed in oysters *M*. *gigas* exposed to the toxic algae *Alexandrium minutum*: contamination in numerous animals remains in the low range of the global distribution data, whereas a minority accumulated the largest amounts [45,46]. Fernandez et al. [47] proposed that this interindividual difference could be due to physiological differences in the feeding performances, digestion rate, and excretion costs. Haberkorn et al. [45] demonstrated that the interindividual variability of PSP bioaccumulation in *M*. *gigas* was positively correlated to the opening duration during the exposure period.

In the present work, significant behavioral changes were observed between the two conditions during the 14 days of exposure. Oysters exposed to cargo ship noise spent more time closed compared to the animals not exposed to ship noise. The difference was concentrated during their active period, which was the day time. In addition, oysters not exposed to ship noise exhibited a higher number of partial-closures than those exposed to ship noise. Partial-closures are partial valve openings and closings that are associated with the back and forth movements of bulk water, which replenishes the water in the pallial cavity. The physiological role of the valve must be taken into consideration. Indeed, the strategy developed by water breathers to ensure their ventilatory activity has been summarized by Massabuau and Abele [48]. The basic idea is that ventilation is a limiting step. The branchial cavity, herein referred to as the pallial cavity, must be viewed as an antechamber in which the water renewal is controlled and slowed when compared to the bulk water. The consequence is that the gills, and the blood inside, face a relatively confined milieu that receives low oxygenation and is hypercapnic. In bivalve mollusks, a decreased quantity and/or amplitude of these partial closures would maintain lower Cd concentrations in the pallial cavity, and under such conditions, a metal concentration gradient will develop in the boundary layer between the gill surface and the bulk-inspired water. Consequently, a decreased diffusion rate from the bulk water to the hemolymph should exist in the gills, limiting the contamination process. Therefore, we propose that, in presence of cargo ship noise, as the valve-opening duration decreased during the daytime and the number of partial-closures also decreased, the gills should be less irrigated by bulk water and thus less exposed to metal uptake. In addition, the excreted metabolic CO_2_ should build up in the branchial cavity, leading to an acidification of water pH in the boundary layers [49,50]. The Free-Ion Activity Model and the Biotic Ligand Model [51,52], which were proposed to predict how free metals interact with external biological membranes, suggest less cadmium uptake and toxicity. However, a prediction is rather difficult, as the problem the oysters face is a balance between more free ions and competition in the gills between the Cd^++^ and H^+^ for binding sites [53]. These ideas were discussed by Campbell [26] to explain how hypoxia and the associated hyperventilation might increase metal contamination in water breathers. We propose to apply them to the present situation and to the analysis of physiological mechanisms explaining the impact of ship noise at larger scale.

### Gene expression

Few studies have reported an induction of oxidative stress under noise exposure. In mice, Cheng et al. [54] observed an increase in the oxidative stress in the encephalic region associated with the lemniscal ascending pathway after they exposed animals to 80 dB re 20 μPa sound for 2 h/day for 6 weeks. Samson et al. [55] also reported a significant effect of noise exposure on free radical scavenging enzymes in the brain of white strain male albino rats. Exposed to acute or chronic white noise at 100 dB re 20 μPa sound for a period of 4 h per day, the animals showed a significant increase in their levels of antioxidant enzymes. The present work also suggests the existence of an oxidative stress under the condition cadmium plus ship noise as an upregulation of *sod2* and *mdh* are reported. Shi and Gibson [56] already shown that oxidative stress can induce an increase in *mdh* gene expression. Without ship noise, and in the presence of Cd alone, the expression of *sod2*, *sod1*, *gpx*, and *mdh* did not change. Cadmium does not directly induce ROS production [57], but it can induce oxidative stress indirectly through different pathways [58]. The absence of enhancement of the oxidative stress defense mechanism at T_14_ in presence of Cd alone suggests either weak oxidative stress or an already fixed problem. In this view, the data show that the *cat* gene coding for the catalase, an enzyme responsible for converting H_2_O_2_ to H_2_O and O_2_, was downregulated more with Cd alone than with Cd plus the cargo ship noise. This larger downregulation agrees with the above dataset, which fits well with a low or absent oxidative stress and higher MT proteins levels in the cells. However, an alternative explanation is that *cat* could also be downregulated through a direct action of Cd [59,60]. Pierron et al. [28] reported a repression of the *cat* gene in the gills of the glass eel (*Anguilla anguilla*) exposed to [Cd]w 10 μg∙L^-1^ during 14 days in brackish water (salinity 0.2 ‰).

Metallothioneins (MT), proteins with a high affinity to metal ions, are considered to play an important role intracellularly in controlling the bioavailability of essential metals and in the protection against the toxicity of nonessential metals, participating in their detoxification. Cadmium is one of the strongest inducers of *mt* gene expression [61]. The accumulation of cadmium in the gills of oysters under both conditions induced a significant overexpression of the *mt2* gene, with significant induction factors (20–27). However, no correlation was observed when a paired analysis of the cadmium concentrations and *mt2* gene expression levels were studied under both conditions. The latter is in accordance with the result obtained by Gonzalez et al. [62] for the zebrafish *Danio rerio* exposed to [Cd]w = 1.9 and 9.6 μg∙L^-1^.

The comparison we report between the expression of *mt2* in the presence or absence of cargo ship noise showed that the median distributions of *mt2* expression were not significantly different (Kruskall-Wallis test) but that the variances were different (Levene test). The later was due to an absence of the higher expression values in the presence of cargo ship noise. The expression of acetyl-coA carboxylase, used to synthetize fatty acids, showed the same pattern of distribution, with all values skewed to the left (Fig 5). Otherwise, Fig 4 shows an under-expression of acetyl-coA carboxylase in the presence of ship noise, indicating a decrease in fatty acid synthesis and lipogenesis. Triglyceride lipase did not change, indicating an absence of enhanced lipolysis. We argue in this work that cargo ship noise exposure would have a depressant impact on the physiology and activity of oysters. The need for energy production is expected to decrease in such a situation, which is in accordance with a decreased expression of acetyl-CoA carboxylase. Food particle sorting in suspension-feeding bivalves is partly based on a recognition mechanism mediated by the lectins contained in the gill mucus [63]. Homolog of these lectins was identified in *M*. gigas (*clec1*) and their highest expressions were not observed in the presence of cargo ship noise but only with cadmium and without ship noise (Fig 5). A lower need for that protein in oysters exposed ship noise correlated to a lower feeding activity is suggested. To summarize this paragraph, the presence of ship noise in addition to cadmium brings some oxidative stress, a lower feeding activity, a decrease of fatty acid synthesis, lipogenesis and lectin synthesis, in comparison to the condition without cargo ship noise. Taken together, these findings fit well with the depressant effect shown by the behavioral analysis and the decrease in growth rate.

### Growth rate

One of the prominent effects of anthropogenic noise exposure is the impact on the growth rate. Oysters exposed to noise showed a low rate of growth compared to oysters without cargo ship noise. Under ambient noise, oysters presented an averaged index of shell growth by approximately 60% in comparison to their congeners. Importantly, this difference cannot be directly attributed to Cd because the oysters that are growing more quickly are also those that are more contaminated. Lagardère [64] observed the same effect of noise on the brown shrimp *Crangon crangon*. Shrimp reared in tanks with a noise level of 30 dB higher than the controls and in a frequency range of 80 to 400 Hz, exhibited a significant reduction in growth. In the present experiment, the growth slow down measured in oysters exposed to cargo ship noise may be partly explained by a reduction in food intake, as their valves were open less often, and the bulk of evidence suggest that the oysters hypoventilated.

## Conclusion

Present data show a depressant action of cargo ship noise on oyster *M*. *gigas* valve opening duration, valve activity, and ventilation, as well as disturbances in fat metabolism. It will be of greatest interest to complete it with more results obtained at diverse sound pressure intensities, during longer times of exposure and by means of field research. But as they stand the data confirm previous reports on a decrease in invertebrate activity and show an unexpected result in terms of metal bioaccumulation processes and growth rates. Cargo ship noise exposure limits Cd contamination and is associated with a slowdown in growth, unraveling a potentially massive risk in terms of ecosystem productivity.

## Supporting information

S1 File Cargo noise sample from Santander port (WAV). (WAV)

S1 Database All data base. (XLSX)
